# Hypoxia-Ischemia Disrupts Directed Interactions within Neonatal Prefrontal-Hippocampal Networks

**DOI:** 10.1371/journal.pone.0083074

**Published:** 2013-12-20

**Authors:** Marco D. Brockmann, Maja Kukovic, Michael Schönfeld, Jan Sedlacik, Ileana L. Hanganu-Opatz

**Affiliations:** 1 Developmental Neurophysiology, University Medical Center Hamburg-Eppendorf, Hamburg, Germany; 2 Department of Neuroradiology, University Medical Center Hamburg-Eppendorf, Hamburg, Germany; Charité University Medicine Berlin, Germany

## Abstract

Due to improved survival rates and outcome of human infants experiencing a hypoxic-ischemic episode, cognitive dysfunctions have become prominent. They might result from abnormal communication within prefrontal-hippocampal networks, as synchrony and directed interactions between the prefrontal cortex and hippocampus account for mnemonic and executive performance. Here, we elucidate the structural and functional impact of hypoxic-ischemic events on developing prefrontal-hippocampal networks in an immature rat model of injury. The magnitude of infarction, cell loss and astrogliosis revealed that an early hypoxic-ischemic episode had either a severe or a mild/moderate outcome. Without affecting the gross morphology, hypoxia-ischemia with mild/moderate outcome diminished prefrontal neuronal firing and gamma network entrainment. This dysfunction resulted from decreased coupling synchrony within prefrontal-hippocampal networks and disruption of hippocampal theta drive. Thus, early hypoxia-ischemia may alter the functional maturation of neuronal networks involved in cognitive processing by disturbing the communication between the neonatal prefrontal cortex and hippocampus.

## Introduction

Hypoxic-ischemic encephalopathy (HIE) occurs in 1-6/1000 live full-births. Its long-term outcome, ranging from intact survival to death, depends on the severity of intrauterine/neonatal insult [1]–[3]. While children with severe HIE develop cerebral palsy as well as major cognitive and sensorimotor deficits, the sequelae are less prominent after a mild or moderate insult. At the opposite end of the severity scale, children with mild HIE appear almost indistinguishable from non-injured controls [4], [5]. Improved medical care in recent years has resulted in decreased mortality and morbidity after a hypoxic-ischemic episode (HI) and increased emphasis on the neurodevelopmental outcome of children with mild/moderate HIE. They predominantly develop high-prevalence/low-severity dysfunctions, such as subtle learning difficulties, abnormal executive and mnemonic functions, which are more pronounced as the children grow and face heavier cognitive demands [6]–[10]. Two mechanisms have been proposed as underlying substrate for mild/moderate HIE: abnormal architectural patterns in gray matter and subtle disruption of interregional connectivity [11]–[13]. The decreased white matter density in the frontal gyrus, miswired neural circuitry between the prefrontal cortex (PFC) and limbic brain areas as well as hippocampal atrophy are of particular interest in the context of HIE-related cognitive disabilities through life [14], [15].

Complex interactions within neuronal networks centered on the PFC and hippocampus (HP) are necessary for information storage and transfer during cognitive tasks [16]–[18]. The PFC receives strong monosynaptic projections from the intermediate and ventral HP [19], [20]. The functional communication between the two regions follows the same directionality and relies on the entrainment of prefrontal-hippocampal networks in oscillatory rhythms [21], [22]. Remarkably, the drive from the HP controls not only the behaviorally-relevant functions of the adult PFC, but also its development. We have previously shown that in rodents the neonatal, but not pre-juvenile prefrontal firing and gamma-band network entrainment critically depend on the hippocampal oscillatory activity [23]. The absence of the hippocampal drive during the neonatal period disturbed the emergence of mnemonic abilities at juvenile age [24].

In light of these findings, the neonatal stage of development (i.e. first postnatal week) appears as a “critical period” for the maturation of rodent prefrontal-hippocampal networks. Consequently, the impact of insults, such as a HI episode, at this age might have a dramatic outcome, perturbing the functional communication within neuronal circuitry that is involved in cognitive processing. To test this hypothesis, it is necessary to induce the HI injury at an early stage of neonatal development, before the onset of directed oscillatory coupling within prefrontal-hippocampal networks. For this, we investigated an immature rat model of the HIE that mimics the HI-induced injury in a third trimester human fetus or preterm child with high prevalence of asphyxia and circulatory collapse [25], [26], (www.translatingtime.net). We combined imaging and electrophysiology *in vivo* with immunohistochemistry to assess the degree of injury and characterize the HI-induced impairment of oscillatory coupling and directed interactions within neonatal prefrontal-hippocampal networks.

## Materials and Methods

All experiments were performed in compliance with the German laws and the guidelines of the European Community for the use of animals in research and were approved by the local ethical committee of the city Hamburg (94/08, 111/12, 132/12). All efforts were made to minimize animal suffering and the number of animals used. Pregnant Wistar rats were obtained at 14–17 days of gestation from the animal facility of the University Medical Center Hamburg-Eppendorf, housed individually in breeding cages with a 12 h light/12 h dark cycle and fed *ad libitum*.

### Hypoxia-ischemia Model

Since rodents are not prone *per se* to hypoxic-ischemic injury during pregnancy or at birth, the HI episode was mimicked by unilateral ligation of the common carotid artery (CCL) followed by exposure to hypoxic environment. We aimed at monitoring the impact of HI on the entire developmental period of directed communication from HP to PFC during the first postnatal week. For this, the Rice-Vannucci model [27] was modified by performing the manipulation as soon as possible after birth instead of at postnatal day (P) 7. All pups considered for this study received the HI episode at P2, because the mortality rate is lower for pups manipulated at P2 than at P1 and the injury patterns are similar at both ages [28]. The mortality rate after a HI episode at P2 was 13.9%. The investigation was performed on a total of 108 remaining rats from 34 litters. Each litter was culled at birth to 14 pups. Due to sex-dependent differences in the outcome of neonatal hypoxia-ischemia, all experiments were conducted in male pups, which, similar to male human infants, have stronger behavioral and cognitive deficits after a HI episode [29], [30]. The pups were anesthetized with isoflurane (5% in O_2_ for induction, 2–3% in O_2_ for maintenance), which does not induce ischemic tolerance, because of its short time of action (<12 min) [31]. A midline incision was made in the neck and the right common carotid artery was dissected, isolated from the jugular vein and vagus nerve and permanently ligated. Pups were returned to the dam for 1–2 h for recovery. Subsequently, rats with unilateral CCL were exposed to preheated 5% O_2_/95% N_2_ mixture delivered at 5.3 l/min by placing them for 120–200 min into chambers floating in a water bath at 37°C. The body temperature of the pups was kept constant at 35°C. The duration of the hypoxic episode was variable. The premorbid appearance of pups and rarely the induction of tonic clonic seizures (n = 2 pups) were used as reliable indicators for the termination of hypoxic episode. Sham-operated animals received the same operation without CCL or hypoxia and were used as controls.

### Magnetic Resonance Imaging (MRI) and Diffusion Tensor Imaging (DTI)

MRI and DTI were performed using a dedicated small animal MR scanner at 7.0 T (ClinScan, Bruker, Ettlingen, Germany). High resolution images were acquired using a receiver only (4 channels) head array coil suitable for mice and rat pups. During imaging rat pups were anesthetized with a gas mixture containing pure oxygen at a flow of 500 ml/min and 1–2% isoflurane that was applied via a vaporizer system (Föhr Medical Instruments, Seeheim-Oberbeerbach, Germany). The respiratory rate (∼20/min) was monitored using a small animal vital sign monitor (SA Instruments, Stony Brook, NY). At defined time points (three hours, five-six days) after the HI episode, axial 2D diffusion weighted (DWI) and T2-weighted turbo spin echo (TSE) images were acquired to detect the presence of injury. Image parameters for the DWI sequence were set as follows: repetition time (TR)/echo time (TE) = 8000/34 ms, bandwidth (BW) = 2790 Hz/pixel, b values = 0, 500 and 1000 s/mm^2^, echo planar imaging (EPI) factor = 128 with parallel acquisition (PA) acceleration factor = 2, number of averages = 2, matrix size = 128×128, field of view (FOV) = 20×20 mm^2^. Twenty slices with 0.8 mm slice thickness and 0.2 mm gap between slices were acquired in 146 s. Image parameters for the T2-weighted TSE sequence were set as follows: TR/TE = 5352/57 ms, BW = 100 Hz/pixel, turbo factor = 7, matrix size = 256×256, FOV = 20×20 mm^2^. Thirty two slices with 0.4 mm slice thickness and 0.1 mm gap between slices were acquired in 396 s.

3D time-of-flight (TOF) angiography was performed in oblique coronal-axial slice orientation to allow sufficient inflow of fresh spins. Image parameters were set as follows: TR/TE = 18/3.7 ms, flip angle (FA) = 25°, BW = 150 Hz/pixel, matrix size = 192×192 and FOV = 20×20 mm^2^. Five image slabs containing 24 slices each (slice thickness 0.12 mm and 25% overlap) were acquired. Acquisition time for the TOF was 336 s. MRI measurements of the cerebral blood flow were obtained using pulsed arterial spin labeling (ASL) with following sequence parameters: inversion time = 1800 ms, labeling time = 700 ms, saturation stop time = 1600 ms, TR/TE = 2400/14 ms, BW = 1985 Hz/pixel, EPI factor = 72 with PA acceleration factor = 2, matrix size = 72×72, FOV = 20×20 mm^2^. Fifty pairs of control and labeling measurements with twenty slices (0.5 mm slice thickness and 1.2 mm gap between slices) were acquired in 247 s.

DTI was performed with following sequence parameters: b-values = 0 and 1000 s/mm^2^, 12 different diffusion weighting directions, 2 averages, TE = 43 ms, TR = 13000 ms, BW = 2790 Hz/pixel, echo planar imaging (EPI) factor = 80 with partial Fourier factor = 5/8, matrix size = 128×80, FOV = 20×20 mm^2^. Twenty four slices with 0.4 mm slice thickness without slice gap were acquired.

Turbo spin echo images were used to determine the brain, hemisphere, and lesion volume for each pup. Mean, axial and radial diffusion maps as well as fractional anisotropy maps were used to quantify the hippocampal projections to the PFC. Regions of interest (ROIs) were manually defined after visual inspection of the T2-weighted TSE or DTI data and quantified with MRIcro software for volumetric image analysis and FDT (FMRIB's Diffusion Toolbox) for projections tracking, respectively. One investigator performed the imaging of all rats without knowing the treatment they received (sham-operation or HI).

### Recording Protocols

Under light urethane-anesthesia (1g/kg; Sigma-Aldrich, Taufkirchen, Germany), the head of the pup was fixed into the stereotaxic apparatus (Stoelting, Wood Dale, IL) using two metal bars fixed with dental cement on the nasal and occipital bones, respectively. The bone over the PFC and HP was carefully removed by drilling holes of less than 0.5 mm in diameter. Removal of the underlying dura mater by drilling was avoided, since leakage of cerebrospinal fluid or blood damps the cortical activity and single neuronal firing (I. Hanganu-Opatz, personal observations). The body of the animals was surrounded by cotton and kept at a constant temperature of 37°C by placing it on a heating blanket. After a 20–40 min recovery period, multielectrode arrays (Silicon Michigan probes, NeuroNexus Technologies, Ann Arbor, MI) were inserted perpendicularly to the skull surface into the PFC until a depth of 3 mm, and at 20° from the vertical plane into the HP at a depth of 2–2.5 mm. The electrodes were labeled with DiI (1,1′-dioctadecyl-3,3,3′,3′-tetramethyl indocarbocyanine, Invitrogen, Darmstadt, Germany) to enable the reconstruction of electrode tracks in the PFC and HP in post-mortem histological sections. Two silver wires inserted into the cerebellum served as ground and reference electrodes. Miniature earphones placed under the pup’s body were sensitive enough to detect the smallest visible movements of the limbs as well as the breathing of pups during recordings.

Simultaneous recordings of the local field potential (LFP) and multiple unit activity (MUA) were performed from the PFC and HP using one-shank 16-channel Michigan electrodes (0.5–3 MΩ). The recording sites were separated by 50 or 100 µm in vertical direction. The recording sites covered the prefrontal sub-divisions cingulate cortex (Cg) and prelimbic cortex (PL) (Van Eden and Uylings, 1985) and the CA1 area in the intermediate/ventral HP. Both LFP and MUA were recorded for at least 2400 s at a sampling rate of 32 kHz using a multi-channel extracellular amplifier (Digital Lynx 4S, Neuralynx, Bozeman, MO) and the corresponding acquisition software (Cheetah).

### Behavioral Analysis

Sham-operated rats and pups with mild/moderate as well as severe outcome of HI were used for the investigation of neonatal behavioral abilities. Each litter was culled at birth to max 14 pups and included both males and females in a constant sex-ratio (3∶2) to avoid sex-based maternal behavioral biases [32]–[34]. To minimize the influence of circadian rhythms, all behavioral tests were conducted during the light phase of circadian cycle. One investigator performed all behavioral testing to prevent inter-observer variability due to different handling of pups.

Pups were tested according to a modified Fox battery [35] for their reflexes and overall growth every second day from P2 to P8. Weight (g), body and tail length (cm) were quantified. Reflexes were scored as follows:

- *Righting reflex*: time (s) until the pup turned over with all four feet on the ground after being placed on its back;
- *Forelimb grasping reflex*: positive (retraction of the paw or grasping of the toothpick) or negative reaction (i.e. no paw movement) when the forepaw was touched with a toothpick;
- *Cliff aversion reflex*: time (s) until the pup withdraws from the edge when the snout and the forepaws are placed over the edge of a table.

### Histology and Immunohistochemistry

Neonatal rats were deeply anesthetized with 10% ketamine (aniMedica, Senden-Bösensell, Germany)/2% xylazine (WDT, Garbsen, Germany) in NaCl (10 µl/g body weight, i.p.) and perfused transcardially with 4% paraformaldehyde (PFA) dissolved in 0.1 M phosphate buffer, pH 7.4. The brains were removed and postfixed in the same solution for 24 h. Subsequently, coronal slices were sectioned at 14–100 µm on a freezing microtome, stored at −80°C and used for different staining protocols.

For the reconstruction of DiI-labeled electrode tracks into the PFC and HP, the slices were stained with Hoechst 33342 dye (1∶1000, Invitrogen, Eugene, OR), air dried, coverslipped with Fluoromont and examined using the 380 and 568 nm excitation filters of the Imager M1 microscope (Zeiss, Oberkochen, Germany).

For post-mortem histological assessment of the injury, the slices were stained for cresyl violet (Merck, Darmstadt, Germany). The thickness of the PL, S1, V1, and CA1 area of the HP were determined for the ligated and non-ligated hemispheres.

For quantification of astrogliosis, slices including the PFC, S1 and HP were stained for glial fibrillary acid protein (GFAP). The slices were pre-incubated for 1 h with PBS-containing 2% normal horse serum, 1% albumin from bovine serum and 0.2% Triton X-100 to block unspecific staining. Incubation with the rabbit anti-GFAP IgG (1∶1000, Promega, Madison, WI) overnight at 4°C was followed by treatment with the secondary antibody Alexa Fluor 568 donkey anti-rabbit IgG for 2 h. Sections were rinsed in PBS several times, counterstained with Hoechst 33342 dye, air-dried, coverslipped with Fluoromont and examined using the 380 and 568 nm excitation filters of the microscope. The intensity of GFAP staining has been quantified by determining the mean and relative pixel density for red staining using Adobe Photoshop CS4. All photographs were adjusted for brightness and contrast using Adobe Photoshop CS4.

### Data Analysis

Data were imported and analyzed off-line using custom-written tools in Matlab software version 7.7 (Mathworks, Natick, MA).

#### Characterization of neonatal patterns of oscillatory activity

To detect the oscillatory events, the raw data were filtered between 4–80 Hz or between 100–300 Hz using a Butterworth three-order filter. The detection and classification of oscillatory events were performed as previously described [23], [36]. Briefly, discontinuous slow oscillations in theta-alpha frequency band were detected as deflections of the LFP exceeding five times the baseline SD. The baseline SD was measured after narrow band filtering of time windows lacking oscillatory activity. Only discontinuous slow events lasting >100 ms, containing at least three cycles and being not correlated with movement (twitches) were considered for analysis. Gamma episodes superimposed on slow events were detected by eye and confirmed by time-frequency plots. Spindle bursts (SB) were classified according to previously described criteria [37], [38]. The presence of short gamma episodes with an amplitude >25 µV on at least one third of the cycles in intermittent slow oscillations led to classification of these events as nested gamma spindle bursts (NG) [23]. Intermittent oscillatory events, SB and NG in the PFC as well as theta bursts in the hippocampal CA1 area, were analyzed in their occurrence (defined as the number of bursts per min), duration, max amplitude (defined as the voltage difference between the maximal positive and negative peaks), and dominant frequency. Time-frequency plots were calculated by transforming the FP events using Morlet continuous wavelet
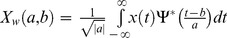
where the wavelet functions are



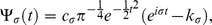


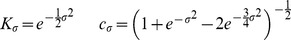



Under the restriction 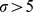 the central frequency of the Morlet wavelet has been chosen as 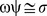.

Minimal and maximal intensities in power were normalized to values between 0 and 1 and were displayed in dark blue and red, respectively.

#### Assessment of correlation between discontinuous oscillatory patterns at different recordings sites

Several methods were used to analyze only co-occurring prefrontal and hippocampal oscillations (i.e. prefrontal oscillations starting within 3 s before or after the hippocampal oscillations). In the first instance, the difference between the onset of discontinuous events in the PFC and of hippocampal theta bursts was calculated. Prefrontal and hippocampal oscillations were termed simultaneous if their onset difference was <13 ms. Secondly, maximal coherence coefficients of FP were calculated for simultaneously recorded prefrontal-hippocampal events using the hippocampal signal as reference. As spectral measure of correlation between two signals across frequencies, the coherence was calculated from the cross-spectral density between the two signals and normalized by the power spectral density of each [39]. The computation was performed according to the formula
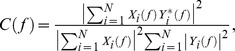
where 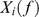 and 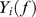 are the Fourier transforms of the signals *x* and *y* for the *i* data segment at frequency *f*, and * indicates the complex conjugate. The computations were carried out using the magnitude-squared coherence function (MATLAB) based on Welch’s averaged periodogram method (non-overlapping 0.5 s time window, frequency resolution 1 Hz). Cross-correlation and coherence values ranging from 0 (events are uncorrelated) to 1 (events are perfectly correlated) were averaged.

#### Spectral analysis and Granger causality

The analysis of directed interactions between simultaneously recorded prefrontal and hippocampal activity was based on the concepts of Wiener-Granger causality [40], [41] applied to autoregressive (AR) models of the data. Statistically, for two simultaneously measured time series, one series can be defined as causal to the other if the second series can be better predicted by incorporating past knowledge from the first one. If the prediction error for the second time series at the present time is reduced by including past data from the first time series in the linear regression model, then the first series can be defined as having a causal or driving influence on the second time series. The previously described analysis [42], [43] and the corresponding toolbox BSMART [44] were modified to fit the long single trial data that are not event-related. The data were divided in short segments of 1 s to avoid non-linear power time series [23]. Briefly, each pair of signals at time *t* were denoted by **X** *_t_* = (*x_1t_*, *x_2t_*)^T^, where T represent the matrix transposition. Considering that the data can be described by the following AR model:
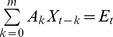
where **E** *_t_* is a temporally uncorrelated residual error series with covariance matrix Σ, and **A**_k_ are 2×2 coefficient matrices to be estimated from the data, we determined the model order *m* by the Akaike information criterion [45]. For all data *m* = 14 was chosen to have optimal spectral resolution and a minimum of overparamerization. Under these conditions the spectral matrix can be evaluated as



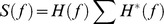
where the asterisk denotes matrix transposition and complex conjugation, and



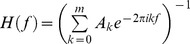
is the transfer function. The power spectrum of channel *l*, which is either 1 or 2 is given by S_ll_(f). The Granger causality spectrum from *x_2t_* to *x_1t_* is defined as shown previously [46], [47]



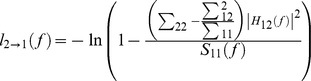
which can be interpreted as the proportion of *x^’^_2t_* causal contribution to the power of the *x_1t_* series at the frequency *f* (in this study theta band). Reciprocally, the causality spectrum from *x_1t_* to *x_2t_* can be obtained by switching the indices 1 and 2 in the above equation. Statistical significance for Granger causality was assessed by using the random permutation approach [47] to build a baseline null-hypothesis distribution. We considered for analysis a large number (between 3000 and 5000) of time segments recorded on two channels in the PFC and HP and we assumed that data from different time segments are independent from each other. Consequently, randomly pairing the time segments from one channel with those from the other for a large number of permutations (n = 100) produces a distribution of causality spectra corresponding to the null hypothesis.

#### Spike sorting and analysis

The raw signal was firstly high-pass filtered (>407 Hz). The threshold for detection of multiple unit activity was individually set depending on the geometry of the recording site. As detailed previously [48] the stored signals were sorted into similar waveform shapes using the Offline Sorter software (Plexon, Dallas, TX). For depicting the valid waveforms in 2D/3D space a combination of features (including the first three principal components, peak-to-peak voltage amplitudes) was chosen. Shapes of detected waveforms were visually inspected to exclude background noise. A group of similar waveforms was considered as being generated from a single neuron if it defined a discrete cluster in a 2D/3D space and exhibited a refractory period (>1 ms) in the interspike interval histograms. Assignment of spikes as being generated by excitatory or inhibitory neurons, as performed in adults is not feasible in neonatal/pre-juvenile pups due to the absence of reliable differences in spike shape during development. The quality of separation between identified clusters was assessed by four different statistical measurements: the classical parametric F statistic of multivariate analysis of variance (MANOVA), the J3 and PseudoF (PsF) statistics and the Davies-Bouldin validity index (DB) [49], [50]. The values of statistical testing ranged between 0.016 and 0.048 for MANOVA, 1.16 and 1.97 for J3, 228.56 and 1009.42 for PsF, and 0.283 and 0.693 for DB.

#### Statistics

Data in the text are presented as mean ± SEM and displayed as bar diagrams. Statistical analyses were performed with SPSS 15.0/Systat software (SPSS GmbH, Munich, Germany). All values were tested for normal distribution by the Kolmogorov-Smirnov test, except where their low number (n<10) precluded reliable testing. For normally distributed values paired or unpaired t-test was used. For a low number of values or not normally distributed values non-parametric tests (Mann-Whitney-Wilcoxon test) were used. Significance levels of p<0.05 (*), p<0.01 (**) or p<0.001 (***) were detected.

## Results

**Figure 1:**
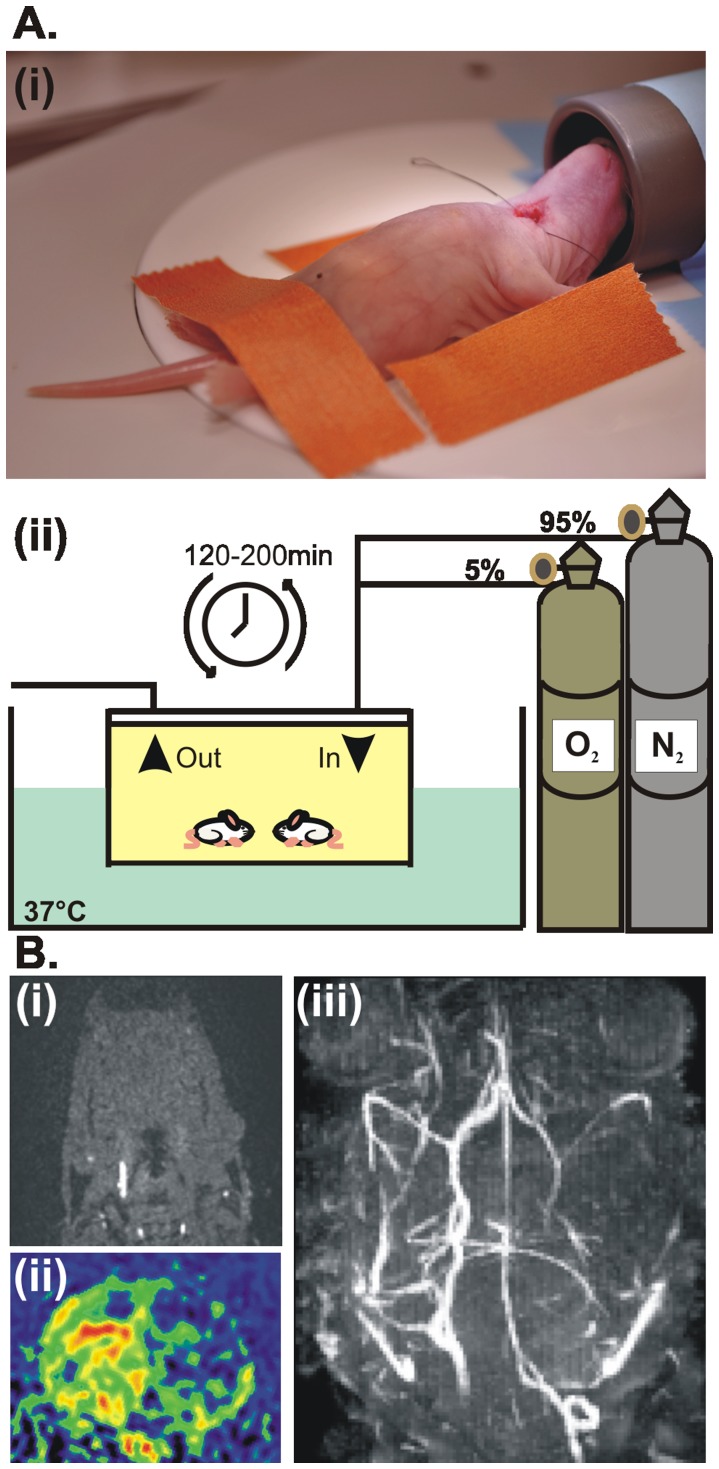
Experimental protocol (modified Rice-Vannucci model) for inducing and confirming the HI insult in P2 pups. (A) Ligation of the right common carotid artery in isoflurane-anesthetized pups (i) was followed by maintenance into the hypoxic chamber (5% O_2_/95% N_2_) at 37°C for a variable period of time (120–200 min) (ii). (B) Confirmation of the HI episode at P2 by angiography and arterial spin labeling. (i) TOF angiogram in axial section at subcranial level demonstrating missing blood flow of the right common carotid artery. (ii) Maximal intensity projection of whole TOF data set showing diminished or missing blood flow of cerebral arteries. (iii) ASL imaging in coronal section maps perfusion deficits in the ligated (right) hemisphere.

We examined the structural and functional effects of an early HI episode in postnatal day (P) 7–8 male rats that received an unilateral common carotid artery ligation (CCL) followed by a hypoxic episode at P2 (n = 56 pups) (Figure 1A). This modified Rice-Vannucci model of neonatal HI [27] allowed monitoring of the early structural and functional impact of injury on the directed prefrontal-hippocampal communication that is initiated at P3 and lasts until the beginning of the second postnatal week [23]. Unilateral interruption of the cerebral blood flow as result of successful CCL was confirmed a few hours after the HI episode by arterial spin labeling (ASL) [51] and time-of-flight magnetic resonance angiography (TOF) [52]. Reduced vascularization and perfusion in the ligated hemisphere as well as normal blood flow in the non-ligated hemisphere were identified immediately after the injury (<3 h) by assessing the maximum intensity projections in coronal and axial sections (Figure 1B).

### Early HI Causes Either Severe or Mild/moderate Structural Injury in Neonatal Rats

**Figure 2:**
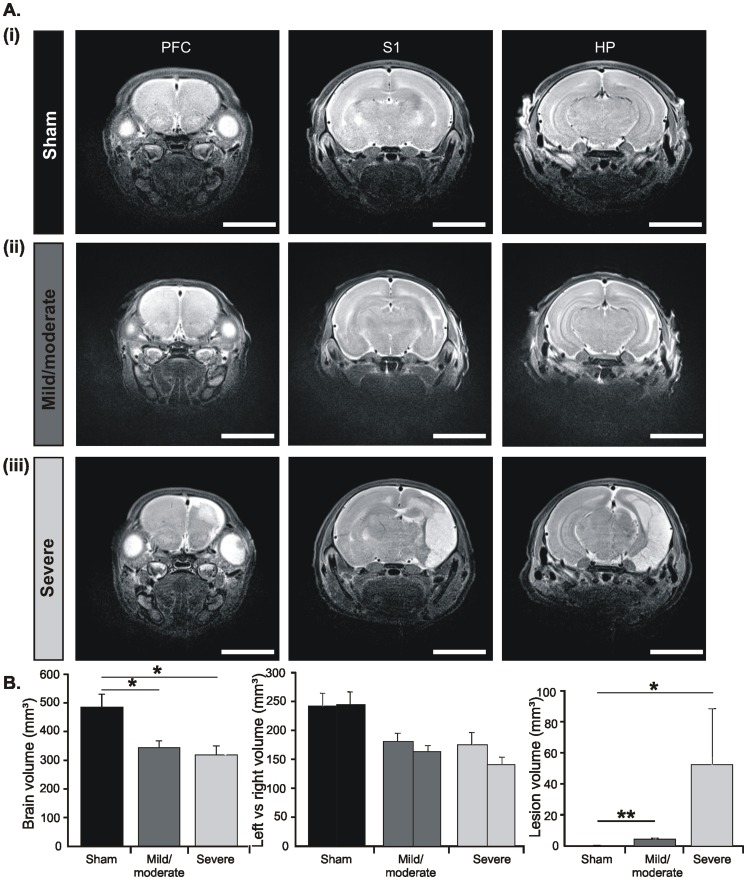
Morphological assessment of the HI injury *in vivo*. (A) Representative T2-weighted MR images in the coronal plane of P7 brains including the PFC (left), S1 (middle), and intermediate HP (right) from a sham-operated pup (i) as well as from pups with mild/moderate (ii) and severe (iii) injury after a HI episode at P2. Sham operation or CCL was performed on the right side. Note the extensive HI-induced lesion (edema/necrosis) over the entire ligated hemisphere of severely injured pups and the small lesion confined over the primary sensory cortices in pups with mild/moderate injury. Scale bars correspond to 6 mm. (B) Bar diagrams displaying the volume of HI-induced infarction (left), the total brain volume (middle) and the volume of the right and left hemispheres (right) averaged for sham-operated pups (n = 4, black bars), pups with mild/moderate outcome (n = 7, dark gray bars) and severely injured pups (n = 3, light gray bars) at P6-7. Data were displayed as mean ± SEM.

Since a HI episode may have variable outcome, we assessed the severity of HI injury at the end of the first postnatal week by three different means. First, T2-weighted magnetic resonance imaging (T2-MRI) revealed an increased signal intensity in the ligated but not the non-ligated hemisphere of P6-7 rats after a HI episode, whereas no signal intensity change was detected in sham-operated pups (Figure 2A). The volume of hyperintense injury varied across rats and allowed the classification of pups with a mild/moderate outcome (n = 7) and pups with a severe outcome (n = 3). The body weight of pups at the time of HI episode did not correlate with the magnitude of lesion (data not shown). In severely injured rats the lateral ventricle appeared enlarged and the lesion mainly affected the neocortex, especially the somatosensory and visual cortices, as well as the HP, while infarction spared the subcortical nuclei. In contrast, pups with mild/moderate outcome showed no T2-MRI intensity increase over the HP and PFC and only a minor lesion confined to the sensory cortices. Correspondingly, the volume of lesion was significantly (p<0.05) smaller in mild/moderate pups (4.2±0.86 mm^3^, n = 7) than in rats with severe outcome (52.14±36.22 mm^3^, n = 3) (Figure 2B). Moreover, a HI episode led to an overall reduction of the brain volume to 344.12±24.7 mm^3^ after a mild/moderate injury and to 315.23±35.31 mm^3^ after severe injury. While the volume of both hemispheres decreased in all injured pups, the effect was more pronounced for the ligated hemisphere and for pups with severe outcome (Figure 2B).

**Figure 3:**
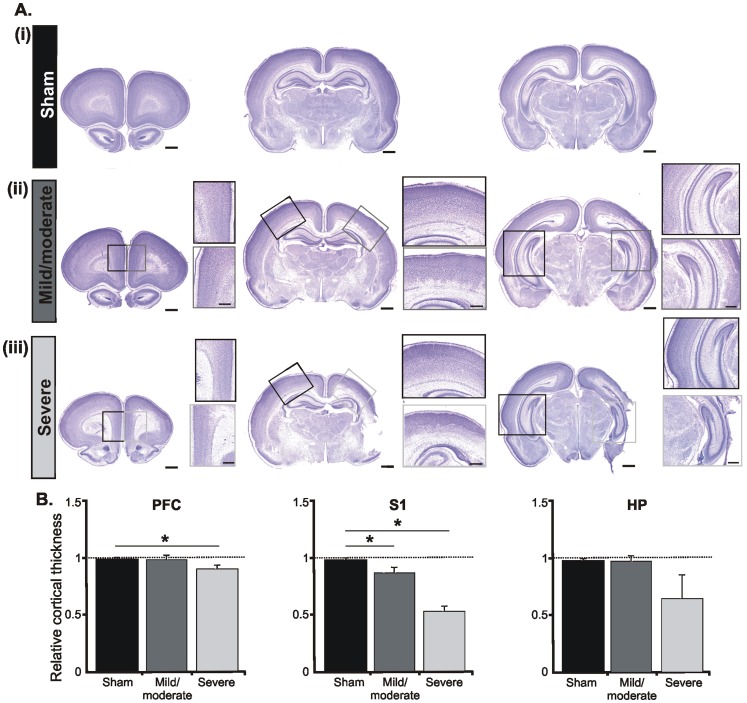
Morphological assessment of the early HI injury *post mortem*. (A) Cresyl violet stained coronal sections at the level of the PFC (left), S1 (middle), and intermediate HP (right) from sham-operated pups (i) as well as from pups with mild/moderate (ii) and severe (iii) injury. Sham operation or CCL was performed on the right side. Scale bars correspond to 1 mm. Insets, higher-magnification photographs from the boxed areas displaying the impact of HI on the cortical architecture of the ligated PFC, S1 and HP (gray boxes) when compared with the non-ligated hemisphere (black boxes). Note the prominent cell loss in the lower layers of the S1 and the hippocampal atrophy as markers of severe injury after a HI episode. Scale bars for insets correspond to 500 µm. (B) Bar diagram displaying the relative cortical thickness of the PFC, S1 and CA1 area of the intermediate HP. The values are normalized to the corresponding regions in the left (non-ligated) hemisphere and averaged for sham-operated pups (n = 5, black bars), pups with mild/moderate outcome (n = 5, dark gray bars) and severely injured P7-8 pups (n = 5, light gray bars). Data were displayed as mean ± SEM.

**Figure 4:**
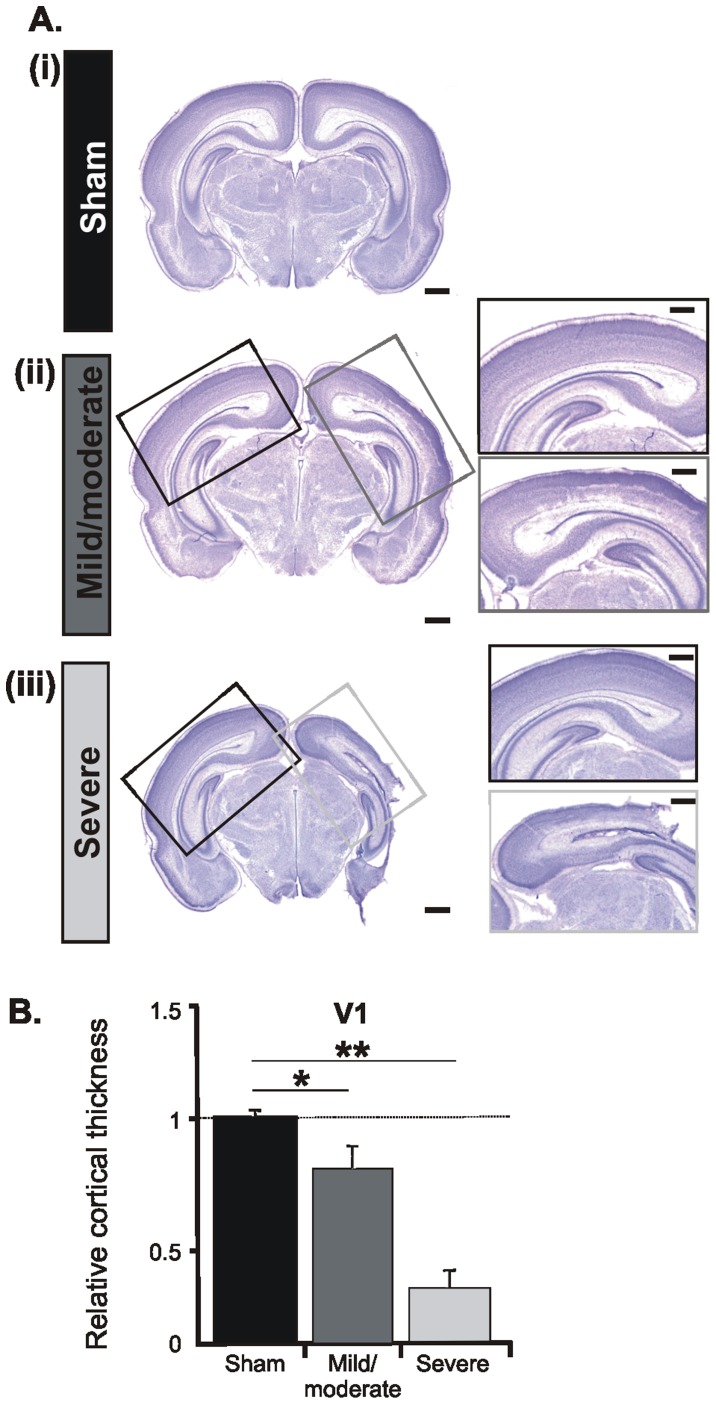
Morphological assessment of the HI injury at the level of V1 *post mortem*. (A) Cresyl violet stained coronal sections at the level of the V1 from P8 sham-operated pups (i) as well as pups with mild/moderate (ii) and severe (iii) injury. Sham operation of CCL was performed on the right side. Scale bars correspond to 1 mm. Insets, higher-magnification photographs from the boxed areas displaying the impact of HI on the cortical architecture of the ipsilateral V1 (gray boxes) when compared with the contralateral hemisphere (black boxes). Note the prominent cell loss in the lower layers of the V1 after a HI episode. Scale bars for the insets correspond to 500 µm. (B) Bar diagram displaying the relative cortical thickness of the ligated V1. Values are normalized to the corresponding area in the non-ligated hemisphere and averaged for all 5 sham-operated (black), 5 pups with mild/moderate outcome (dark gray) and 5 severely injured (light gray) P7-8 pups. Data were displayed as mean ± SEM.

The second experimental approach histologically confirmed the differential impact of an early HI episode on the brain of neonatal rats (n = 15). According to the size of the MRI-detected infarction, the magnitude of cortical cell loss in cresyl violet-stained coronal sections graded the injury in severe and mild/moderate (Figure 3, Figure 4). Pups with severe injury showed necrotic areas, cystic formation, extreme thinning of the entire cortex, and in extreme cases partial absence of sensory, perirhinal and entorhinal cortices (Figure 3A). Shrunken cells with pyknotic nuclei, as indicative of cell death, are present in the majority of cortical areas. In relationship to sham-operated pups (n = 5) or the non-ligated hemisphere the thickness of the PFC in the ligated hemisphere decreased by 10% and of the HP by 34%. The most prominent effect was observed in the primary somatosensory (S1) and visual (V1) cortices, where the neocortical thickness was reduced by more than half (Figure 3B, Figure 4). As previously reported, the cell loss principally affected the lower cortical layers and the remnants of the subplate [28]. In contrast, rats with mild/moderate injury showed minimal, if any, cell loss in the PFC (relative thickness ligated vs. non-ligated hemisphere: 0.98±0.04) and HP (relative thickness ligated vs. non-ligated hemisphere: 0.98±0.05). Larger cell loss took place only in S1 and V1, where cell-free columns expanded over the deeper cortical layers (Figure 3Aii, Figure 4Aii, insets).

**Figure 5:**
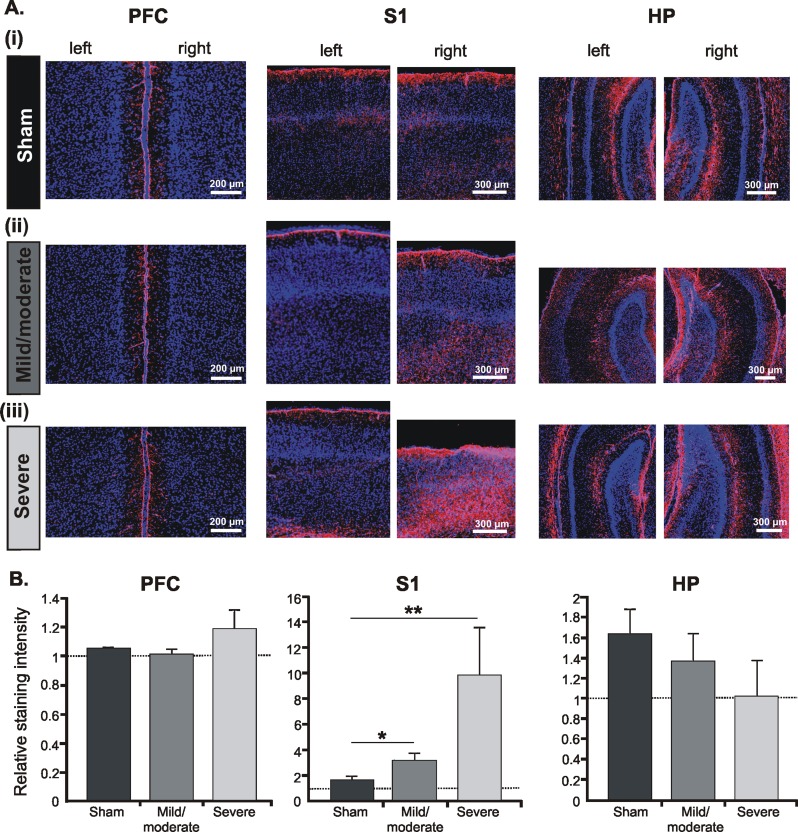
Assessment of astrogliosis after an early HI episode. (A) Nuclear Hoechst 33342 (blue) combined with GFAP immunofluorescent staining (magenta) of coronal sections at the level of the left (non-ligated) and right (ligated) PFC (left), S1 (middle), and intermediate HP (right) from P7 sham-operated pups (i) as well as pups with mild/moderate (ii) and severe (iii) injury. Note the dramatic increase in the astrocyte reactivity in the lower layers of the S1 after mild/moderate or severe injury and the absence of astrogliosis in the PFC and hippocampal CA1 area. (B) Bar diagrams displaying the relative intensity of GFAP-staining in the right (ligated) PFC, S1 and CA1 area of the intermediate HP when normalized to the corresponding regions in the non-ligated hemisphere. Data were displayed as mean ± SEM.

The third method to quantify the severity of injury focused on the HI-induced astrogliosis. In addition to affecting neuronal integrity, HI causes astrocyte proliferation, which is meant to contribute to the survival and repair of hypoxic-ischemic tissue [53], [54]. Immunostaining of glial fibrillary acid protein (GFAP), which is a marker for astrocyte scarring, revealed augmented proliferation of astrocytes in rats experiencing a HI episode, but not in sham-operated pups. The degree of proliferation was region-dependent and corresponded to the severity of injury (Figure 5A). The magnitude of astrogliosis dramatically increased in the ligated S1 of severely-injured pups to 9.86±3.7 fold when compared to rats with mild/moderate injury (3.15±0.6) or sham-operated pups (1.7±0.3). In contrast, no significant astrogliosis was present in the PFC and HP of rats with mild/moderate injury (Figure 5B). Even pups with severe outcome showed minimally increased astrocyte proliferation in the PFC and no astrogliosis at all in the HP.

In line with these results, the outcome of an early HI episode on the neonatal cortical morphology can be classified as either mild/moderate with minimal cell loss, infarction and astrogliosis in the sensory cortices, but not in the PFC and HP, or as severe with large infarction, prominent cell loss and massive astrocyte proliferation all over cortical areas.

### Early HI does not Impair the Somatic Development, Feeding Abilities and Reflexes of Neonatal Rats

**Figure 6:**
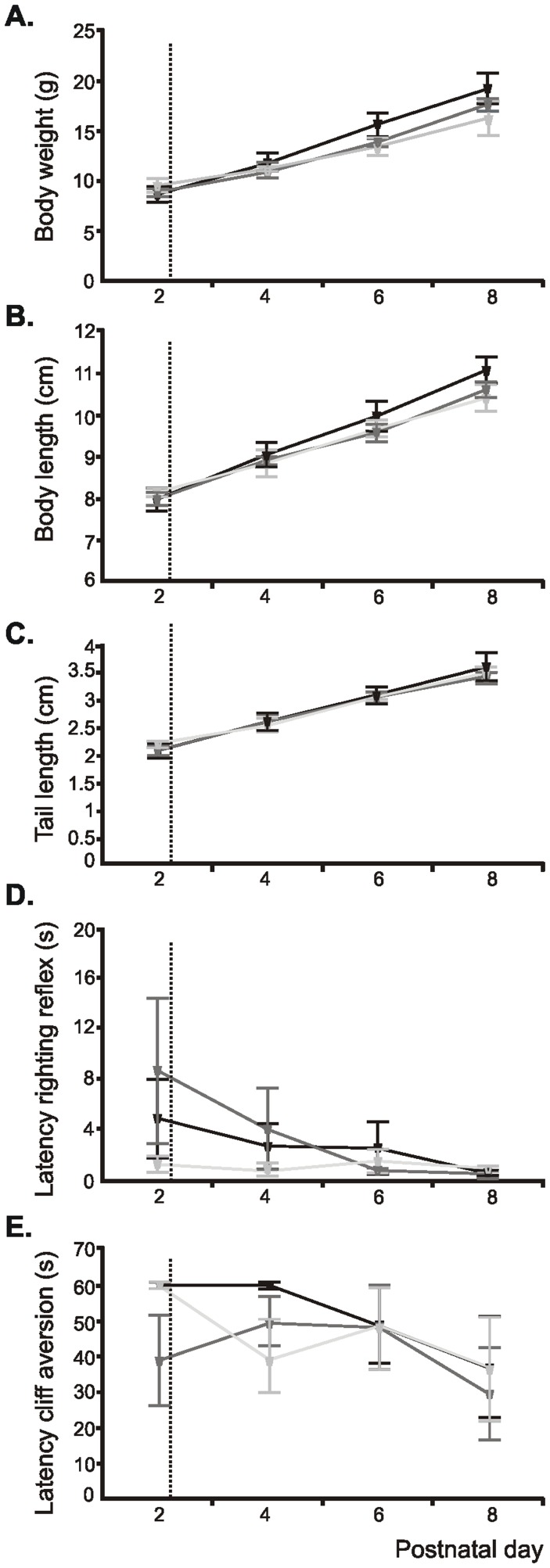
Consequences of an early HI episode on the somatic development and reflexes of neonatal rat pups. Developmental profile of somatic growth [body weight (A), body length (B), tail length (C)] and reflexes [righting reflex (D), cliff aversion reflex (E)] in sham-operated pups (n = 5, black) as well as in pups with mild/moderate outcome (n = 5, dark gray) and severely injured pups (n = 5, light gray). The dotted line marks the HI episode. Data are displayed as mean ± SEM.

Pups experiencing an early HI insult may be physically disabled with retardation of somatic development, feeding abilities, and reflexes. In this case, the brain activity and functional communication within neuronal networks will be equally impaired, not as direct effect of HI injury, but as by-product of retarded somatic growth. To decide whether pups experiencing an early HI insult are physically healthy, we quantified over the entire first postnatal week the somatic development and reflexes of sham-operated rats as well as of pups with mild/moderate and severe HI outcome. All three groups showed progressive increase in body weight from 8.66±0.8 g in sham-operated (n = 5), 8.86±0.42 g in mild/moderate (n = 5), and 9.58±0.64 g in severely-injured pups (n = 5) at P2 to 19.2±1.42 g, 17.6±0.6 g, and 16.24±1.72 g respectively, at P8. The body and tail length augmented similarly (Figure 6A–C). Although being below the significance level, the differences in the somatic development between HI-injured and sham-operated pups increased with ongoing maturation and were more prominent in pups with severe outcome. Moreover, pups experiencing a HI episode at P2 had normal reflexes, neither the righting nor the cliff aversion or grasping reflex showing disturbed developmental dynamics (Figure 6D, E).

The normal somatic development and reflexes of pups experiencing a HI episode at P2 may be due to better tolerance of injury at early stages of development. Previous studies reported retardation of somatic growth and neurological reflexes after a HI episode at P4 or P7 [55], [56]. Thus, early HI does not induce non-specific impairment or delay of physical neonatal development.

### Mild/moderate HI Injury Disrupts Gamma Entrainment of Prefrontal Networks but not Hippocampal Theta Activity

**Figure 7:**
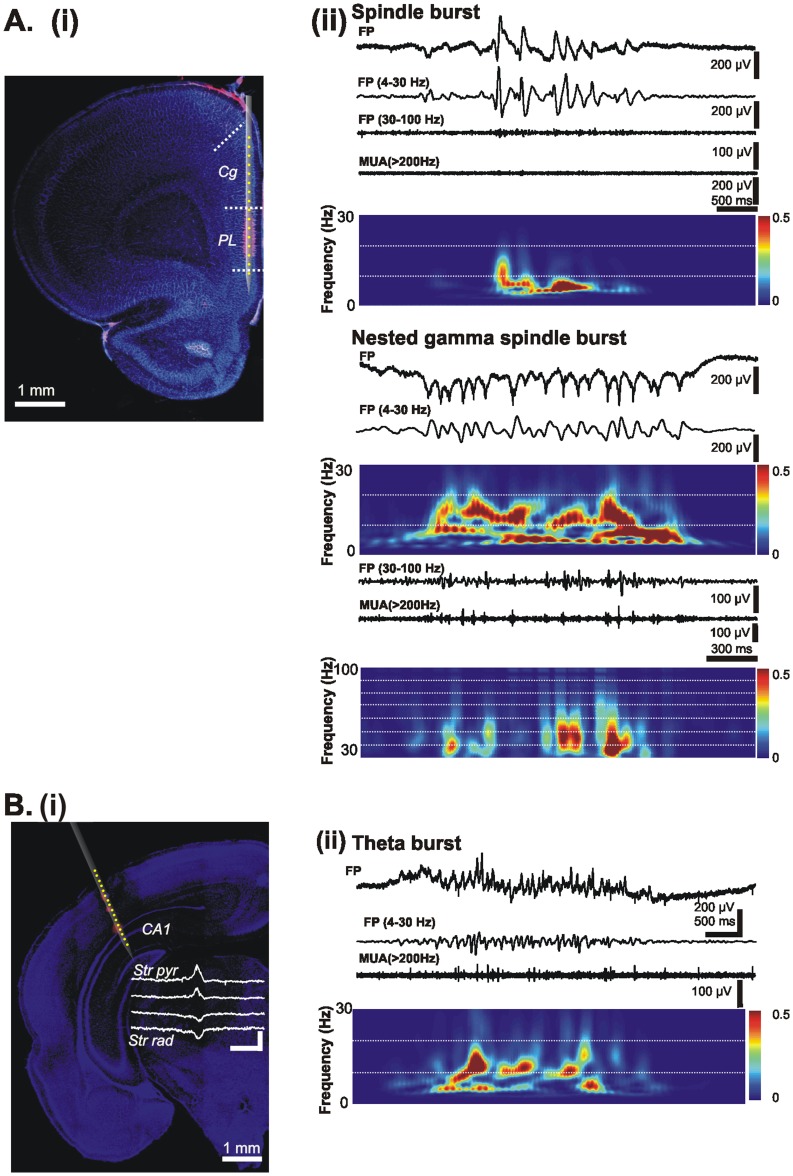
Patterns of oscillatory activity in the neonatal PFC and HP *in vivo*. (A) (i) Digital photomontage reconstructing the location of the DiI-labeled recording electrode (magenta) in the PFC of a Nuclear Hoechst 33342-stained 100 µm-thick coronal section (blue) from a P7 rat. Yellow dots mark the 16 recording sites covering the Cg and PL. (ii) Characteristic SB and NG displayed before (top) and after band-pass (4–30 and 30–100 Hz) filtering (middle), and the corresponding MUA after 200 Hz high-pass filtering. Note the presence of prominent NG-characteristic gamma episodes that are accompanied by spike discharge and the low firing during SB. The upper color-coded wavelet spectrum reveal the main frequency of SB within theta band and of NG within theta-alpha band, whereas the nested gamma episodes appear as periodic high power spots on the bottom wavelet spectrum of NG. (B) Digital photomontage reconstructing the location of the DiI-labeled recording electrode (magenta) in the hippocampal CA1 area of a Nuclear Hoechst 33342-stained 100 µm-thick coronal section (blue) from a P7 rat. Yellow dots mark the 16 recording sites covering the CA1 area and the S1. Scale bars correspond to 200 µV and 100 ms. Inset, prominent sharp-waves reversing between Str pyr. and Str rad. (ii) Characteristic CA1 theta burst displayed before and after band-pass (4–30 Hz) filtering and the corresponding MUA after 200 Hz high-pass filtering. Color-coded frequency plot shows the wavelet spectrum of the FP at identical time scale.

The absence of gross morphological injury in the PFC and HP after a HI episode with mild/moderate outcome raised the question whether the two cortical areas are particularly resistant to insult or HI mainly affects their function by impairing the oscillatory entrainment within prefrontal-hippocampal networks. To experimentally address this question, we examined the patterns of network activity in the prelimbic subdivision (PL) of the PFC and in the CA1 area of the intermediate/ventral HP (Figure 7), which are known to strongly interact both during development and in adulthood [23], [57], [58]. For this, we performed extracellular recordings of the LFP and MUA in P7-8 sham-operated rats (n = 7) and pups with mild/moderate HI injury (n = 6) *in vivo*. The extensive neurodegeneration and infarction over the HP precluded recordings in pups with a severe HI insult.

**Figure 8:**
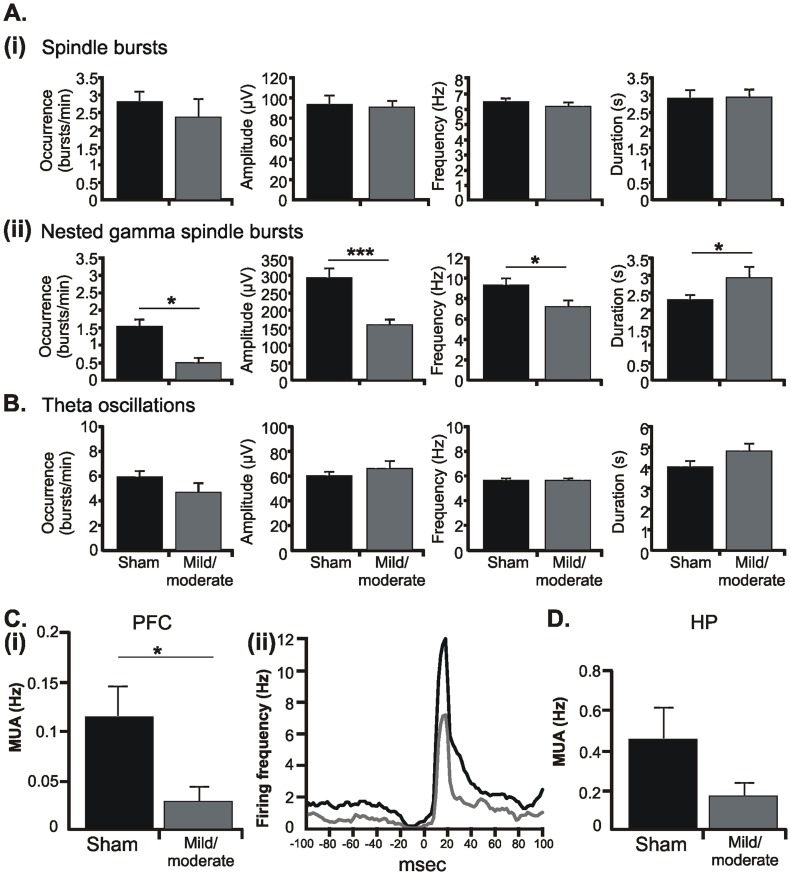
Consequences of HI on the oscillatory network activity and firing of neonatal PL and HP. (A) Bar diagram displaying the occurrence, amplitude, main frequency, and duration of SB (i) and NG (ii) recorded from the PL of 6 P7-8 sham-operated rats (black) and 6 pups with mild/moderate HI injury (dark gray). Note the selective effect of HI on the NG. (B) Bar diagram displaying the occurrence, amplitude, main frequency, and duration of theta bursts recorded from the CA1 area of the intermediate HP in 6 P7-8 sham-operated rats (black) and 6 pups with mild/moderate HI injury (dark gray). (C) HI-induced changes in the occurrence and temporal distribution of firing in the PL. (i) Bar diagram displaying the firing rate of prelimbic neurons in 7 P7-8 sham-operated pups and 6 rats with mild/moderate outcome. (ii) Representative histogram correlating MUA with the onset (time lag 0 ms) of gamma episodes. Values are averaged from 5 sham-operated pups (black) and 3 pups with mild/moderate injury (dark gray). Note that neurons from both sham-operated and HI-injured pups fire preferentially ∼20 ms after the onset of gamma episodes. (D) Bar diagram displaying the firing rate of CA1 hippocampal neurons in 7 P7-8 sham-operated and 6 HI injured pups. For all diagrams data are displayed as mean ± SEM.

Both sham-operated and injured pups showed similar discontinuous patterns of oscillatory activity in the PFC as those previously reported for non-manipulated rats [23]. The dominant pattern of prelimbic activity was an intermittent spindle-shaped field oscillation with main frequency in theta band that we defined as spindle burst (SB) (Figure 7Aii). It was accompanied by intermittent theta-alpha bursts with superimposed fast gamma episodes that we previously characterized as nested gamma spindle bursts (NG). While mild/moderate HI changed none of the properties of SB (Figure 8A), it strongly affected the NG (Figure 8B). Their occurrence and amplitude decreased from 1.53±0.2 bursts/min and 296.52±26.78 µV in sham-operated pups to 0.48±0.14 bursts/min (p = 0.028) and 158.91±16.55 µV (p = 0.0007), respectively, whereas the duration increased from 2.36±0.13 s to 2.99±0.31 s (p = 0.028). The dominant frequency of NG switched from the alpha (9.46±0.7 Hz) to theta (7.3±0.64 Hz) frequency band.

We previously showed that in non-manipulated rats nested gamma episodes time the firing of prefrontal neurons and therefore, reflect the entrainment of local neuronal networks within PFC [23]. Corresponding to the reduced occurrence of NG, MUA was diminished from 0.11±0.03 Hz in sham-operated pups (n = 7) to 0.03±0.01 Hz after insult (n = 6) (Figure 8Ci). Examination of individual neuronal firing after clustering of spikes revealed that the number of active neurons decreased from 9 in sham-operated pups to 3 neurons in rats with mild/moderate HI. However, the injury did not affect the tight coupling between gamma episodes and MUA, since the prominent peak in their cross-correlogram emerged ∼20 ms after the onset of gamma episode (0 ms time lag) in both groups (Figure 8Cii).

In contrast to the prominent impact of HI on the prefrontal activity, the network activity in the CA1 area of the intermediate/ventral HP was not affected by HI with mild/moderate outcome. Towards the end of the first postnatal week, theta bursts represent the main activity pattern in the HP (Figure 7Bii). Their occurrence was slightly decreased to 4.67±0.72 bursts/min after an early HI insult when compared with sham-operated pups (5.93±0.48 bursts/min), but the change was not statistically significant. Similarly, the amplitude, duration and dominant frequency in theta band did not significantly differ between the two groups of pups (Figure 8B). The mild/moderate HI insult did not modify either the MUA discharge (0.46±0.16 Hz in sham-operated rats, n = 7; 0.17±0.07 Hz in injured rats, n = 6) or the individual firing of clustered neurons (8 in sham-operated rats, 7 in injured rats) (Figure 8D).

These data indicate that in the absence of prominent morphological impairment of the neonatal PFC, its patterns of network activity and neuronal firing were significantly modified by a HI episode with mild/moderate outcome. In contrast, neither the morphology nor the network activity and spiking patterns of the HP were affected by HI.

### Mild/moderate HI Injury Decreases the Coupling Synchrony and Impairs Directed Interactions within Neonatal Prefrontal-hippocampal Networks

**Figure 9:**
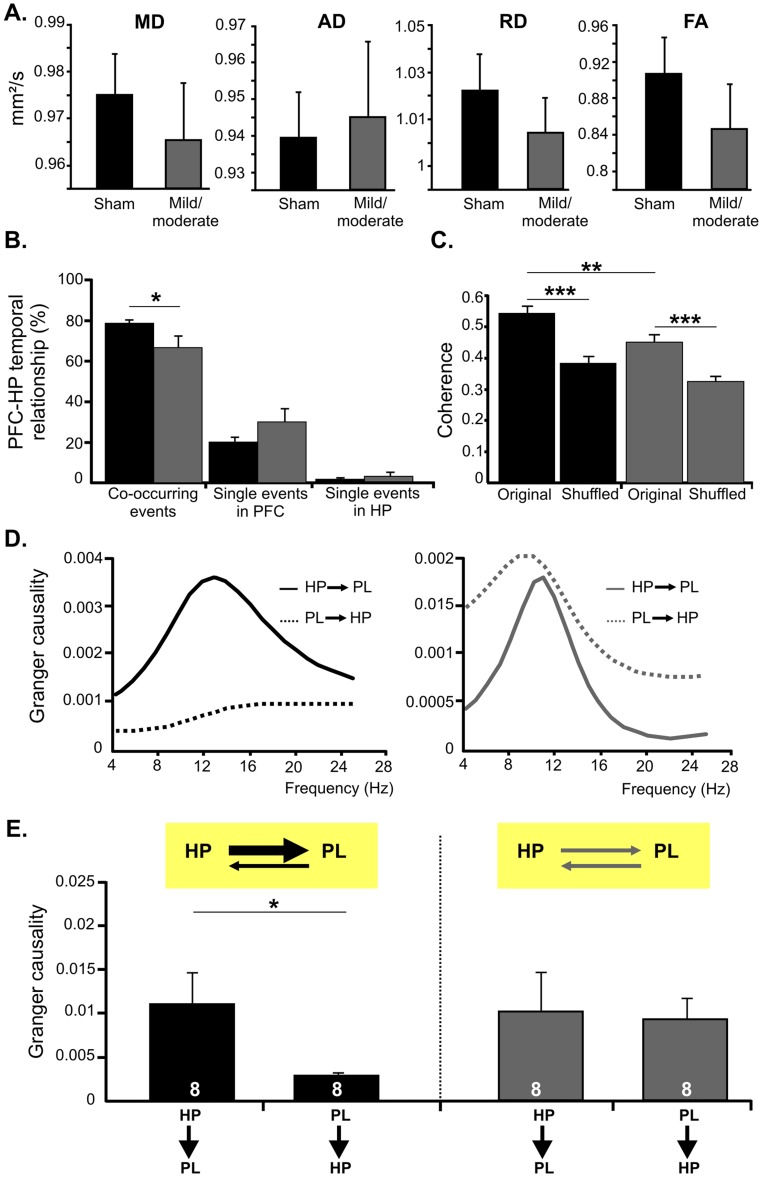
Consequences of HI on the dynamic coupling and directed interactions within neonatal prefrontal-hippocampal networks. (A) Bar diagrams displaying the ratio of MD, AD, RD and the FA for the left and right hemisphere in sham-operated (black) and HI-injured pups (dark gray). (B) Bar diagram (mean ± SEM) displaying the relative incidence of simultaneously occurring vs. one area-restricted prelimbic oscillations and hippocampal theta bursts recorded from 5 P7-8 sham-operated (black) and 5 pups with mild/moderate injury (dark gray). (C) Coherence of prelimbic-hippocampal oscillations calculated for sham-operated rats (n = 5) and pups with mild/moderate injury (n = 5). Bar diagram (mean ± SEM) reveals the significantly higher prelimbic-hippocampal coherence for original than for shuffled data (oscillatory events in the PL and HP occurring at different time points) in both groups of pups. (D) Examples of Granger causality spectra between the PL and CA1 area of a P7 sham-operated (left) and HI-injured pup (right). (E) Bar diagrams (mean ± SEM) displaying the mean Granger causality averaged over significant (p<0.05) pairs of time segments for the interactions PL-HP at the end of the first postnatal week. The number of investigated pups is marked on bars. Insets, schematic representation of the causal relationship between neonatal prefrontal and hippocampal theta activity under physiological and HI conditions.

To determine whether an early HI episode with mild/moderate outcome affects the prefrontal-hippocampal interactions during neonatal development, we used several approaches. First, we assessed the impact of HI on the unidirectional and monosynaptic projections from the ventral HP to the PFC [57]. For this, quantification of the projections by anterograde and retrograde tracing is less suitable due to the variability of tracer’s amount from pup to pup and the transport properties of healthy vs. injured tissue (M. Frotscher, personal communication). Therefore, we aimed at quantifying the prefrontal-hippocampal connections by DTI tracking of sham-operated (n = 6) and HI-injured pups (n = 5). To ensure a good resolution a highfield magnet (7.0 T) was used. Multiple ROIs were defined on the tract of hippocampal afferent fibers [59]. Mean (MD), axial (AD), and radial (RD) diffusion as well as fraction anisotropy (FA) maps were calculated for left and right hemisphere and revealed no significant differences between the two groups of pups (Figure 9A). These data suggest that the anatomical connectivity from the HP to the PFC is not severely injured after a HI episode with mild/moderate outcome.

Second, we examined the temporal correspondence of discontinuous oscillations across the PL and CA1 area of the intermediate/ventral HP in simultaneous recordings from both areas. In sham-operated pups, the majority of hippocampal theta bursts (78.38±1.77%, n = 193 events from 5 pups) occurred within a narrow time window (<3 s) with the prelimbic SB or NG, as previously reported for non-manipulated pups [23]. The mild/moderate HI insult decreased this temporal coupling between the PFC and HP (66.78±5.56%, n = 139 events from 5 pups) and increased the occurrence of temporally-uncoupled region-specific events (Figure 9B).

In the third approach, we confirmed the HI-induced de-coupling of the PFC and HP by assessing the temporal synchrony between the two areas. In sham-operated pups, coherence coefficients for simultaneously occurring oscillations in the PL and HP were relatively high (0.54±0.02, n = 50 events) as measure of the strong synchronization within prefrontal-hippocampal networks (Figure 9C). Moreover, the significantly smaller coherence coefficients for oscillations at shuffled time windows (0.38±0.02, n = 50 events) and the high synchronization between prelimbic LFP and hippocampal spikes argue for a time-dependent genuine coupling between the PL and HP and a limited, if any, contribution of conduction synchrony. The synchronization within prefrontal-hippocampal networks was decreased in pups with mild/moderate injury (coherence coefficient 0.45±0.02, n = 50 events), while still being higher than for oscillations at shuffled time windows (0.32±0.02, n = 50 events).

Due to the symmetric interdependence of coherence, the decreased synchronization between the PL and HP after a mild/moderate HI insult does not offer reliable insights into the impact of injury on the directionality of information flow between the two brain areas. To estimate the effects of HI on the causal strength of prefrontal-hippocampal interactions, Granger causality analysis, a powerful method for studying directed interactions between brain areas [43], [46], was carried out. Pairs of signals filtered in theta frequency band (4–12 Hz) from the PL and HP of sham-operated rats and pups with mild/moderate HI outcome were used for analysis. The causal relationships found between the two regions in sham-operated pups were similar to those previously identified in non-manipulated rats. Since the peak Granger causality values were significantly higher for the HP driving the PL (HP→PL) than for the reciprocal connection PL→HP (n = 8) (Figure 9D, E), the hippocampal theta bursts more strongly drove the prelimbic oscillations than vice versa. The results are in line with the stable coupling between the PL and HP as revealed by coherence analysis and support the driving role of hippocampal theta bursts for the prelimbic oscillations. In contrast, a different causal relationship was found between the neonatal PL and HP of pups with mild/moderate injury. While the peak values of Granger causality for pairs of signals from the PL and HP were still significant in all investigated pups (n = 8), they were similar for both directions of information flow, HP→PL and PL→HP. Thus, the mild/moderate HI injury during the first postnatal week seems to disrupt the directed interactions within prefrontal-hippocampal networks, depriving the developing PFC from the entraining action of the hippocampal theta drive (Figure 9E).

The statistical nature of Granger causality analysis and its limitations [60] require confirmation of Granger-causal relationships by other means. For non-manipulated rats the analysis of spike-timing relationship between prefrontal and hippocampal neurons revealed similar theta-locked prelimbic firing after hippocampal discharge at neonatal age, confirming the results and the applicability of Granger analysis to our data sets. The dramatic decrease of spiking frequency in the PL after the HI insult precluded the confirmation of Granger-causality by cross-covariance analysis of pups with HI insult. However, it is very likely that HI-induced disruption of the hippocampal drive equally disturbs the timing of prefrontal firing.

These results indicate that, even in the absence of major morphological injury, an early HI episode with mild/moderate outcome dramatically perturbs the functional communication within neonatal prefrontal-hippocampal networks.

## Discussion

In the present study, we combined MRI, DTI, and immunohistochemistry with multielectrode recordings *in vivo* to decide whether an early HI episode affects the functional development and interplay of prefrontal-hippocampal networks. This interaction between PFC and HP is initiated during the first postnatal week and accounts for adult cognitive performance [17], [61]. Using a rat model of human HIE during the third gestational trimester, we demonstrate that despite the gross morphological integrity of the PFC and HP as well as of their axonal connectivity after an early HI episode with mild/moderate outcome, the patterns of network activity and the interactions between the two areas are significantly impaired. HI reduced the prefrontal neuronal firing and gamma-band oscillatory entrainment of local networks. Moreover, HI decreased the coupling synchrony within developing prefrontal-hippocampal networks and impaired the theta drive from the HP to the PFC, which previously has been shown to facilitate the functional maturation of the PFC [23], [24], [36]. Thus, disruption of functional communication within prefrontal-hippocampal networks during a “critical” developmental period of directed interactions might act as underlying mechanism of later HI-induced cognitive disability.

### Modeling Different Outcome of HIE in Rats

Taking into account the ethical and technical limitations of investigations in premature human infants, elucidation of the mechanisms responsible for the HIE-related sequelae requires the use of a suitable animal model. While most widely used, the Rice-Vannucci model mimics in seven days-old rat pups the HI injury of a 34 weeks-old human fetus [62], [63]. To model the infant prematurity associated with true intrauterine HI, we modified the Rice-Vannucci model by subjecting younger rat pups (P2) to ligation of the common carotid artery and reduced O_2_ concentration. According to established neurodevelopmental parallels across species [25], (www.translatingtime.net), the first postnatal days in rodents correspond to the second-third gestational trimester in humans. During this time frame key developmental events, such as receptor maturation, astrocyte formation, axonal and vascular maturation, take place and have been identified as critical for the outcome of injury [64]. Equally relevant for the current investigation, the directed functional communication within prefrontal-hippocampal networks emerges at this age [23].

Besides age, several other factors control the extent of HIE-related brain damage. Some of them, such as body temperature, gender, and duration of the hypoxic insult [29], [65], [66], are easily controllable in an animal model. Although we kept them constant in our experimental approach, the injury still had a variable outcome. Since the body weight of rats at the time of injury did not correlate with the size of lesion, the variability of outcome might relate to the position in uterus and the variable branching of circle of Willis [67], [68]. According to the magnitude of morphological changes, the HI-induced injury has been classified as either mild/moderate or severe. We assessed the infarction, cell loss and astrogliosis to confirm this classification. The morphological investigation additionally revealed the different impact of HI on cortical areas and layers. Columnar cell loss and astrocyte proliferation was present in the deeper layers of the primary sensory cortices and their magnitude progressively increased with the severity of injury. The layer-specific injury is mainly caused by cerebrovascular immaturity over the cortical depth [69], [70]. In contrast, the PFC and HP appeared less vulnerable to HI, confirming previous findings [28].

### Mechanisms of HI-induced Abnormal Communication within Neonatal Prefrontal-hippocampal Networks

The results of the present study demonstrate that an early HI episode causing no major morphological impairment still disrupts the functional communication between the neonatal PFC and HP. The developmental stage of this functional disconnection of the two areas coincides with the time window, during which the immature PFC [71] relies on the unidirectional excitatory drive from the HP to initiate and consolidate the assembling of local neuronal networks [23]. In the absence of directed interactions from the HP (sender) to the PFC (receiver) after a HI episode, both the coupling synchrony within prefrontal-hippocampal networks and the activation of local prefrontal networks were diminished.

Three mechanisms may account for the HI-induced functional disconnection of the neonatal PFC and HP. First, the HP is unable to initiate and/or send the excitatory drive to the PFC. This hypothesis is poorly supported by our experimental data. Neither the hippocampal theta bursts nor the neuronal firing in the CA1 area of the HP, which drive the prefrontal activity via synaptic projections, were impaired after the HI episode. The resistance of hippocampal activity to injury might be due to its multiple origin involving brain areas (e.g. medial septum) with different vulnerability to HI insult [72].

Second, the PFC is unable to follow the received hippocampal drive, because HI causes functional modifications of the prefrontal microcircuitry. The profound changes in the patterns of discontinuous oscillatory activity of the neonatal PFC, especially of the superimposed gamma bursts in NG, strongly support this hypothesis. Subplate neurons, a transiently expressed cell type located beneath the cortical layers [73], are particularly vulnerable to an early HI insult [28] and coordinate the emergence of neocortical patterns of oscillatory activity [74]. Since these neurons assist in targeting of corticofugal and corticopetal connectivity and process information from cortical and subcortical areas [75], [76], their selective death after HI profoundly may disturb the maturation of neuronal networks in the PFC. We hypothesize that in the absence of a functional subplate, the ingrowing hippocampal projections lack the necessary relay station for guidance to the final target, the prefrontal layers V–VI [20] and therefore, may be unable to produce activation of local prefrontal networks. Another cellular mechanism accounting for HI-induced impairment of network oscillations may involve the GABAergic neurons in the PFC, the density of which increased after HI [77]. Since the generation of prefrontal gamma bursts critically depends on the activation of GABAergic neurons [36], the imbalance between excitation and inhibition may represent an additional source of abnormal wiring and impaired network activity within the PFC.

Third, impaired or diminished axonal projections, which cannot forward the excitatory drive from the HP to the PFC, might be responsible for the disruption of directed interactions within prefrontal-hippocampal networks. At the developmental stage of injury, an exuberant axonal growth takes place [3], which is preferentially perturbed by HI with mild/moderate outcome. Diffuse axonal degeneration that emerges immediately after the insult [78]–[80] may affect the hippocampal projections to the PFC and theta-modulated communication within prefrontal-hippocampal networks. However, in line with the DTI analysis, it is unlikely that severe impairment of projections from the HP to the PFC accounts for the functional disturbance of network activity. We cannot still exclude that discrete impairment of diffuse hippocampal projections was below the resolution of used magnet and led to similar diffusion and anisotropy maps of sham-operated and HI-injured pups.

Thus, we propose that the disruption of directed communication within neonatal prefrontal-hippocampal networks after mild/moderate HI mainly results from abnormal microcircuitry of the PFC.

### Physiological Relevance of Disrupted Prefrontal-hippocampal Communication for Cognitive Disability after HI Insult

A wealth of studies documented the cognitive disability as well as the academic underachievement caused by a HI insult during early developmental stages [4], [7], [9], [10], [14]. In the absence of massive morphological injury, the mnemonic and executive impairment usually remains unrecognized during early childhood [81] and becomes obvious during the heavier cognitive demands of later stages of development [82]. The crucial function of the PFC in gating of memory, attention, and decision making [83] led the focus of investigation toward the impact of HI on the prefrontal integrity. However, at the macroscale of non-invasive approaches that are suitable for human infants, solely severe prefrontal pathology has been linked to cognitive deficits. For example, the reduction of tissue volume in the PFC was proportional with the performance decrease in working memory tasks [84].

In an animal model of HI insult we could easily assess whether subtle changes of functional communication within PFC or prefrontal-limbic networks are caused by the injury. The perturbed oscillatory activity and disturbed entrainment of neonatal prefrontal-hippocampal networks that we reported here may delay and/or impair the developmental refinement of connectivity and juvenile processing in the PFC and HP. Consequently, the achievement of mnemonic and executive tasks critically depending on the integrity of this communication may be equally affected by HI. Future studies will decide whether the functional impairment (abnormal activity patterns, synchronization and communication deficits) after an early HI injury persists, increases or is compensated at juvenile stage and to which extent its dynamics correlates with the onset and performance level of cognitive abilities.
